# Can red tourism lead to spiritual transformation? Evidence from tourists visiting the Red Army Long March Xiangjiang Battle Memorial Park

**DOI:** 10.1371/journal.pone.0280920

**Published:** 2023-07-07

**Authors:** Huiling Zhou, Qianru Zhang, Yajun Jiang, Fuyuan Wang

**Affiliations:** 1 College of Tourism and Landscape Architecture, Guilin University of Technology, Guilin, China; 2 Department of Chinese Classics, Hunan University of Science and Engineering, Yongzhou, China; 3 Institute of Geographic Sciences and Natural Research, Chinese Academy of Sciences, Beijing, China; 4 Key Laboratory of Regional Sustainable Development Modeling, Chinese Academy of Sciences, Beijing, China; Universidad Central de Chile, CHILE

## Abstract

The mechanism of spiritual transformation in red tourism plays a key role in facilitating the inheritance of red culture. A survey of 385 tourists of Chinese nationality was conducted to explore the path of red tourism’s influence on tourists’ spiritual transformation. Based on the stimulus–organism–response theory, this paper explores tourists’ environmental perceptions of red tourism activities as special external stimuli, introduces a positive emotion factor, and constructs a path model of red tourism for tourists’ positive emotions based on educational function and cultural identity, which ultimately leads to their spiritual transformation. The results of the empirical tests using structural equation modelling indicated that environmental perceptions had a significantly positive effect on the stimulation of positive emotions, while positive emotions had an indirect effect on spiritual transformation. The research results enhance people’s understanding of the spiritual transformation brought by red tourism and provide management significance for red tourism planning.

## Introduction

Red tourism is an important channel for showcasing the glorious revolutionary history of the Communist Party of China (CPC). In addition, red tourism helps to stably spread core socialist values and advance socialism with Chinese characteristics to a far-reaching extent. Despite that red tourism is a hot topic in China, there are some forms of international tourism activities with similar themes. These countries have taken advantage of the war sites against national aggression and the revolutionary relics that led the national independence movement as tourism resources, with the main purpose of creating destinations with patriotic educational functions [1]. Such forms of tourism normally include communist heritage tourism [2] and dark tourism [3]. Though there are some differences between domestic red tourism and that of other countries, both the purpose of development and construction and the nature of tourism will lead to a deeper spiritual significance. In this respect, their essence and connotation are in consistence. Visiting these sites and monuments, tourists will have a strong sense of patriotism and national pride, especially after visiting the tourist destination, they will cherish peace more and cherish the present happy life [4].

Tourists, inspired by the revolutionary spirit, pay tribute to the revolutionary heroes of the past at historical sites [5]. Internationally, most of the studies on the spiritual significance or value of tourism have explored the dark tourism and communist heritage tourism in the West. Communist heritage sites are closely linked to the reconstruction of local history and the search for a new national identity [5]. Dark tourism can bring learning benefits and spiritual significance. It not only allows tourists to obtain collective identity, but also allows them to gaze at their own position in the world [6–8]. The transformation mechanism of tourist spirit in red tourism destination is generated in the process of internalization of revolutionary spirit. In China, researches on the impact of red tourism mainly focus on values [9–12], education [10, 11, 13], identity [14, 15] and spirit [16, 17]. The unique quality of red culture tourism plays a very important role in stimulating the national spirit of tourists, carrying forward the spirit of red culture (including the tenacious spirit of resistance) and enhancing their cultural confidence in the Chinese nation [13]. According to Liu, the spiritual and educational functions of red tourism activities can encourage tourists to visit red tourism sites, experience red history, and experience spiritual transformation and purification [16]. Yang showed the core value of red tourism is to inherit red culture; In other words, the spirit of red culture is an important carrier of red culture [17]. Therefore, the development of red tourism activities should consider the extraction and dissemination of the red cultural spirit [17].

Although the literature on the spiritual value of red tourism is gradually abundant, there are still some problems. The researches lack the theoretical explanation of the spiritual value of red tourism. In addition, scholars have not made a systematic review on the mechanism of forming the spiritual value of red tourism. Lin found that the phenomena in the revolutionary historical period were not recreated and proliferated, but simply used as the expression of developing red tourism products without considering their spiritual value [18]. The internalization of the revolutionary spirit in the Long March of the Red Army and other red cultural values is the spiritual transformation of tourists when they experience red tourist attractions. The key variables and mechanisms involved in this process need to be further explored. Based on the above analysis, emotional variables are introduced in this paper to explore how the educational function and cultural identity of red tourism generated by tourists’ emotional perception is internalized into their spiritual transformation. Based on the stimulus–organism–response (SOR) model, the research results are of great significance for enriching the study on the value of red spirit tourism, enhancing the vitality of red tourism development in the new era, and promoting the development of high-quality red tourism products.

## Literature review and research hypothesis

### Environmental perception and positive emotions

The conceptual framework of this study is mainly based on the SOR model proposed by Mehrabian and Russell [19]. The SOR model has been widely applied in tourism research [20–22], suggesting that environmental stimuli (S) trigger cognitive and emotional responses of organisms (O), thus triggering corresponding behavioral responses (R).

In contrast to previous studies, this study focuses on the relationship between environment, emotion, and response, namely that environmental stimuli (i.e. environmental stimuli of red tourism) may influence visitors in red tourism (i.e. educational function, cultural identity, and spiritual transformation) through organic emotional responses (i.e. positive emotions). This study uses the SOR model as its conceptual framework to explain how these factors are related.

According to the SOR model of psychology [23], tourists receive stimuli and form experiences from tourism activities, which lead to responses through a series of mental activities [19]. Liu et al. found that tourists first receive stimuli from the external environment before the tourism process evokes certain types of emotions [24]. Red tourism destinations provide tourists with a special external environment with a strong political atmosphere. Li et al. showed that the historical heritage of the CPC is reinterpreted for tourists at specific red tourism sites [25]. Under the stimulation of various activities and environments, tourists’ emotions toward the CPC and their perceptions of Chinese revolutionary history may change [14]. Based on cognitive appraisal, emotional appraisal, and positive emotion expansion–construction theories, Liu et al. confirmed that that red tourism experiences can positively influence tourists’ emotions [26].

Jiang et al. showed that the facilities, services, and surroundings at red tourism sites act as environmental stimuli to trigger emotional and behavioural changes in tourists [15]. By representing the invisible past at red tourism sites and museums, tourists may obtain a reflective view of their contemporary society, consolidate their social perceptions, and experience an authentic perception of their own existence [4]. Following scientific and technological advances, many red tourism sites are now designed to stimulate an emotional response in visitors through their use of sound and spatial design. Red tourism activities may also use technology such as electronic guides and special headphones to help visitors engage more closely with the exhibitions and have immersive experiences [7]. Xie and Peng considered tourism activities to facilitate tourists’ search for pleasure or delight and argued that the basic level of their tourism activities is emotional. The surrounding environment also often plays a subtle role in influencing tourists’ emotions and behaviours [4]. Accordingly, we propose our first hypothesis:

H1: Environmental stimuli have a significantly positive effect on positive emotions.

### Educational function and cultural identity

Cultural identity behaviour refers to citizens’ cultural recognition of a certain cultural environment, which is influenced by their cultural perceptions and emotions and driven by certain motivations [27]. Cultural identity behaviour is also ephemeral, as it manifests as an attitude of recognition of a particular cultural period based on a longitudinal comparison of a national culture over time [28]. Red culture refers to the advanced culture with Chinese characteristics created by proletarian revolutionaries, Marxist intellectuals, and the masses of people under the leadership of the CPC during the New Democracy period (1919–1949) [29]. Luo and Wu showed that there has been a historical breakthrough in the number of people visiting red tourism sites in recent years; thus, red tourism has effectively spread red revolutionary culture [30].

Liu et al. found that compared to other forms of tourism, the positive emotions generated by tourists visiting memorial sites during red tourism activities are complex and include mainly positive emotions (e.g., moved, amazed, shocked) and positive–negative emotions (e.g., sad, sorry, frustrated) [24]. Nawijn and Fricke showed that tourists’ positive and negative emotions while visiting dark tourism sites can have a long-term impact on their behaviour [31]. Red cultural history’s emotional elements should be explored fully in red cultural education, such as the shocking revolutionary spirit and admirable tenacity of revolutionary heroes, in addition to mournful scenes. These elements enrich red culture, which becomes more concrete and more infectious. In this way, red tourism activities increase their educational impact on Students from major universities in China [32].

Tourists make two positive migrations during tourism activities. The first is “two positives make a positive,” that is, positive content and emotions such as the positive effect of revolutionary events and heroes, engage with tourists’ value identification. The second is “two negatives make a positive,” which is similar to the “dark tourism” of red tourism. Dark tourism activities generate negative emotions in tourists based on their “tragic beauty,” which can also stimulate tourists’ emotional feedback and empathy in observing their contemporary lives and value identification [12]. By participating in red tourism activities, tourists perceptually evaluate their external environment in addition to their internal perceptual evaluation of themselves, which evokes individual emotions and ultimately changes their own cognitive attitudes toward individuals for the purpose of learning and education [6]. Red tourism activities provide educational opportunities that promote patriotic and collectivist values [33] and deepen visitors’ political [14], national, and cultural identities [26]. Museums and battlefield sites are not just places where tourists come to receive information and have emotional experiences, but can also be places where tourists obtain knowledge from the offered history [8]. Tourists’ cross-cultural experiences during their travels can create a sense of cultural identity and serve as a source of education [34]. Zhang and Ma clearly showed that educational experiences and cultural identity are highly positively correlated [35]. Accordingly, we propose the following two hypotheses:

H2: Positive emotions have a significantly positive impact on educational function.
H3: Positive emotions have a significantly positive effect on cultural identity.

### Spiritual transformation

The term “spiritual transformation” is derived from the exploration of the spiritual meaning that tourism sites bring to tourists by Zheng et al. [6]. Zheng et al. observed that tourists seek meaning from spiritual aspects of tourism sites that go beyond ordinary tourists’ intentions. This spiritual transformation helps to solidify tourists’ self-identity [6], which is similar to the “transformation” of the impact of red tourism on tourists themselves in the context of their understanding of Chinese culture. The impact of red tourism on tourists is seen not only during their visits to red tourism sites but also in their return to life by revitalizing their faith in the government and sometimes even changing their lifestyle [36]. Spiritual transformation integrates the impact of red tourism on tourists’ ideological values during their experiences at red tourism sites and is the process of internalizing the revolutionary spirit, such as the spirit of the Red Army’s and red cultural values, which is the process of spiritual transformation of the tourist.

Zheng et al. showed that tourists’ positive emotions and positive–negative emotions during tourism experiences can have a direct or indirect impact on their spiritual consciousness [6]. Dark tourism sites such as memorial exhibitions seek to convey a sense of psychological identity to dark tourists by increasing their emotional participation, which ultimately leads to internalization of the sites’ spiritual meaning [7]. Red culture can only become the common spiritual wealth of the Chinese people if they reach a consensus about its value to contemporary society [37]. Red culture is the spiritual home of Chinese communists, a spiritual repository based on cultural value identity. Thus, red culture is the general public’s recognition and trust in their ownership of the Chinese communists’ spiritual culture, expressed as cultural confidence, emotional support, psychological recognition, and behavioural following [37].

Hosseini observed that the importance of dark tourism to the ideology and spirit of Chinese people cannot be measured by its market economic value, but must be measured by tourist sites’ educational significance for tourists’ consciousness [4]. Education plays an essential role in the process of transforming cultural values into spiritual motivation [38]. Through the study of red culture, cultural values can be internalized into conscious ideological leadership and spiritual pursuits [39]. In addition, Zhang and Liu found that neglecting rational discursive cognition also affects the effect of university students’ identification with red culture [40]. Thus, education plays a key mediating role in the process of generating spiritual meaning. Accordingly, we propose the following three hypotheses:

H4: Positive emotions have a significantly positive impact on spiritual transformation.
H5: Cultural identity has a significantly positive impact on spiritual transformation.
H6: Educational function has a significantly positive impact on spiritual transformation.

**Fig 1:**
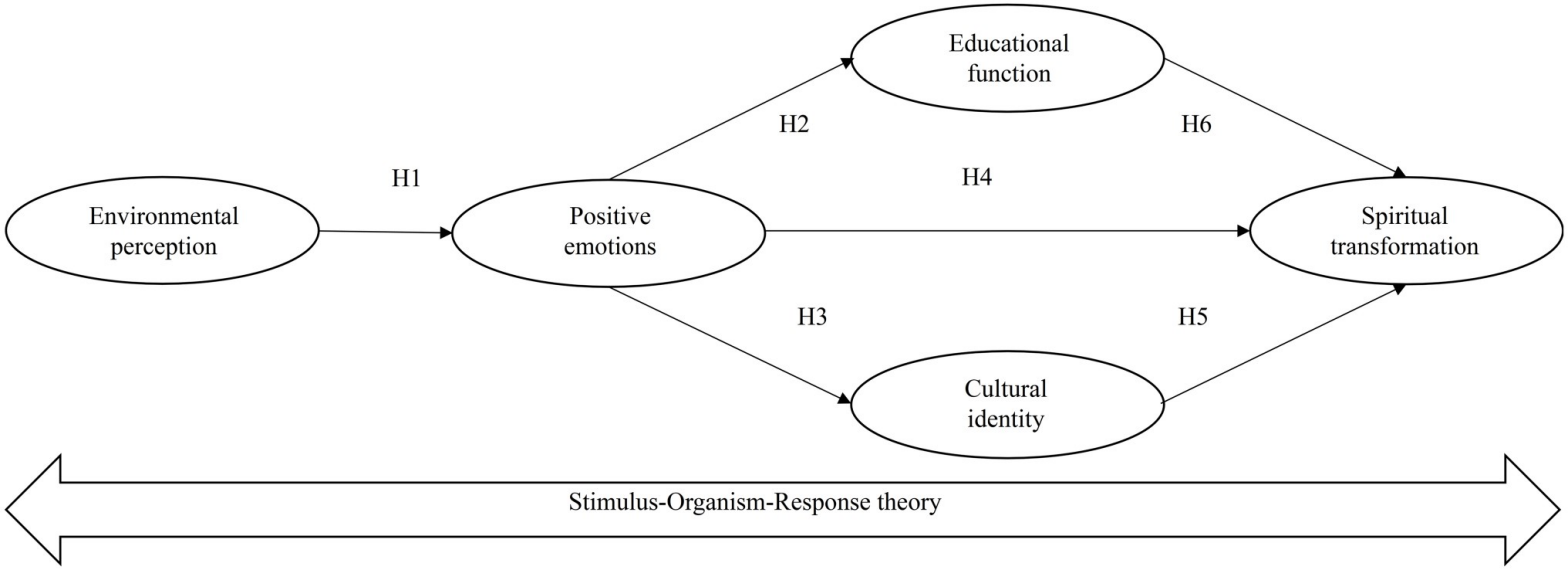
The hypothetical model.

This paper proposes the following hypothetical model (Fig 1).

## Methods

### Data collection

The research uses structural equation modeling (SEM) to analyze valid data from the survey, a multivariate statistical analysis method capable of measuring potential structures identified through factor analysis and evaluating the path of hypothesized relationships between structures. SEM analysis is carried out using a two-stage method. In the first phase, confirmatory factor analysis is used to measure the adequacy of the measurement model. In the second stage, the structural model is evaluated. The goodness of fit index (GFI) is used to evaluate the overall model fit of the measurement and structural model [41].

We distributed our questionnaire in August and October, 2021 because many people travel to tourist destinations during this time of year. Therefore, we generated a broad representation of gender, age, and occupation, among other demographic characteristics. The research has been reviewed by the Academic Committee of the School of Tourism and Landscape Architecture of Guilin University of Technology. During the research, participants were informed of the research contents at the beginning of the questionnaire, then the contents were verbally restated, and participants agreed to and completed the questionnaire after being told about the confidentiality of the research. The questionnaire was distributed to visitors at the exit and near the parking area of the Red Army Long March Xiangjiang Battle Memorial Park. The fieldwork staff observed that some respondents completed the forms too quickly and perfunctorily; these were flagged as invalid questionnaires. Other questionnaire forms that respondents showed a clear tendency to complete quickly were also removed (e.g., 10 consecutive items marked in the same way). Generally, in order to ensure the scientific rationality of structural equation model analysis, the total number of measured indicators is at least 10 times of the sample size [41]. This research uses 19 measures to reflect 5 related potential variables. In this study, 358 valid questionnaires were obtained, which met the requirement of sample size.

The Red Army Long March Xiangjiang Battle Memorial Park is a key exhibition area of the Long March National Cultural Park located in Caiwan Town, Quanzhou County, Guilin City. The Memorial Park is the former site of one of the two major blockade battles during the Xiangjiang Battle, namely, the One Foot Mountain Shop Blockade. The Red Army Long March Xiangjiang Battle Memorial Park is a thematic memorial site showcasing the entire history of the Red Army’s Long March. The site comprehensively and systematically illustrates the entire history of the Xiangjiang Battle and the Red Army’s Long March and focuses on China’s glorious achievements under the strong leadership of the CPC. The exhibition hall at the Memorial Park has two floors, with a total exhibition area of 4,545 square meters displaying 379 pictures, 306 cultural relics, 14 thematic sculpture groups, 28 artworks, 9 scenes, 21 interactive multimedia items, and 1 panoramic exhibition hall. At present, the Red Army Long March Xiangjiang Battle Memorial Park is rated as a national patriotic education demonstration base among the national 4A-level tourist attractions and is designed to facilitate patriotic education, party education, clean government education, and red studies, among other activities.

### Measurement development

Our two-part survey questionnaire used a 7-point Likert scale (i.e., 1 = strongly disagree, 7 = strongly agree). The first part included five measurement items: environmental perception, positive emotions, educational function, cultural identity, and spiritual transformation. Previously developed measurement scales were used to ensure the necessary psychometric rigor. Environmental perception was measured using the scale developed by Chen with four indicators [42]. The scale developed by Liu et al. was used to measure positive emotions and included two indicators [24]. The multi-item scale to measure educational function was adapted from Li et al. and comprised four items [43]. Zhang derived the four indicators used to measure cultural identity from the definition of the concept of red culture and its historical evolution [44]. Finally, spiritual transformation was measured using a set of items adapted from Zheng et al. [6]. The second part of the questionnaire collected respondents’ demographic information, including their gender, age, income, educational level, occupation, and political affiliation.

## Results

### Respondent profiles

Descriptive statistics were obtained using SPSS software (v. 24; IBM SPSS, Inc, Armonk, NY, USA) to create the respondents’ profiles (Table 1). Slightly more women (56.42%) than men (43.58%) completed the questionnaire. The majority (37.15%) of respondents were 19–24 years old, 32.96% were 25–44 years old, 16.76% were 45–64 years old, 11.45% were under 18 years old and 1.68% were over 65 years old. The distribution of the respondents’ various occupations was relatively even. Regarding the respondents’ monthly income, 38.55% had a monthly income of less than RMB 2,000, and the remainder of the respondents had a monthly income between RMB 3,000 and 8,000. Nearly half (48.32%) of the respondents had a Bachelor’s degree, 22.63% had completed high school education, 12.57% had not completed high school education, and 16.48% had a postgraduate degree. A majority of respondents (37.99%) were politically affiliated with the masses. Overall, the sample was relatively evenly distributed and considered suitable for this study.

**Table 1:** Respondent profile.

Categories		Frequency	Percentage (%)
Gender	Male	156	43.58
	Female	202	56.42
Age (years)	Below 18	41	11.45
	19–24	133	37.15
	25–44	118	32.96
	45–64	60	16.76
	65 and above	6	1.68
Occupation	Students	142	39.66
	Institutions	48	13.41
	Companies	81	22.63
	Freelance	77	21.51
	Retirees	10	2.79
Income per month (in RMB Yuan)	Below 2000	138	38.55
	2000–2999	32	8.94
	3000–4999	92	25.70
	5000–7999	58	16.20
	8000 and above	38	10.61
Education	Below high school	45	12.57
	High school	81	22.63
	college	173	48.32
	Graduate school or higher	59	16.48
Political status	CPC	85	23.74
	Preparatory member of CPC	19	5.31
	Communist Young League	117	32.68
	A party other than CPC	1	0.28
	General public	136	37.99

### Analysis of measurement models: Reliability and validity

Before testing the measurement model, we evaluated the internal reliability of the scales using Cranach’s α coefficients (range, 0.74–0.83). All of the coefficient values were above 0.70 and considered acceptable. We then used validation factor analysis to assess the validity of the measurement models. The factor loadings were all above 0.6, except for the guided tour component of environmental perception, where the factor loadings were below 0.60. Based on the result indicating that tourists reported that they did not hire a tour guide to narrate their red tourism activities, we removed this item and retested the modified measurement model. Satisfactory factor loadings were obtained for all measurement items, as they all exceeded the 0.6 threshold at the p<0.001 significance level.

Based on the fit results, the following data were obtained for each of the measurement models: χ2 = 366.938, df = 125, comparative fit index (CFI) = 0.924 > 0.9, and root mean square error of approximation (RMSEA) = 0.074 < 0.08. The results indicated good fit indices for each of the measurement models. The construct reliability (CR) of the five potential constructs (i.e., environmental perception, positive emotions, educational function, cultural identity, and spiritual transformation) were 0.76, 0.82, 0.83, 0.83, and 0.83, respectively, with the CRs of the potential constructs exceeding the recommended threshold of 0.7. The average variance extracted (AVEs) for each construct ranged from 0.51 to 0.61, with AVEs greater than 0.5 being ideal. Therefore, all of the model constructs had acceptable convergent validity (Table 2).

**Table 2:** Measurement model and CFA results.

Variables and indicators	Mean	SD	SL	α	CR	AVE
Environmental perception				0.74	0.76	0.51
The visual impact of the display of group sculptures, firearms and other landscape artifacts is stunning	5.84	1.25	0.66			
The preservation of historical resources such as artifacts and old photographs has contributed to a strong revolutionary atmosphere	6.02	1.09	0.86			
Guided tour signage systems such as timelines and site description boards make it easier for me to understand the story of the revolution	5.97	1.10	0.60			
The guide’s vivid and realistic explanation gave me a deeper understanding of the revolutionary story	5.82	1.34	Delete			
Positive emotions				0.81	0.82	0.61
I Struck by the heroic spirit of the Red Army	6.34	0.95	0.77			
I marveled at the determination and tenacity of the revolutionary fighters	6.32	1.08	0.87			
I feel sad for the tens of thousands of revolutionary heroes who died	6.35	1.06	0.70			
Educational function				0.82	0.83	0.54
Inspired by my patriotic passion	6.41	0.91	0.73			
Reinforced my communist ideals	6.34	1.00	0.69			
Gave me a deeper understanding and recognition of Marxism, Mao Zedong Thought, etc.	6.46	1.12	0.79			
I learned about the revolutionary spirit of hard work and dedication	6.34	1.03	0.75			
Cultural identity				0.83	0.83	0.55
Red culture carries on the spirit of patriotism	6.51	0.86	0.70			
Red culture enhances cultural confidence in socialism with Chinese characteristics	6.44	0.85	0.78			
Red culture nurtures the "Chinese dream" of the revival of the Chinese nation”	6.39	1.01	0.76			
Red culture unites the spirit of national culture	6.47	0.94	0.73			
Spiritual transformation				0.83	0.83	0.55
I will love my country more	6.55	0.85	0.73			
I think young people should be more responsible for the prosperity of our country	6.46	0.93	0.75			
I believe that the Chinese nation will become stronger and stronger	6.59	0.86	0.71			
I think we should always remember history	6.58	0.79	0.79			

We then tested the discriminant validity of our results (Table 3). The correlations between the latent variables were significant, and all of the correlation coefficients were less than the square root of AVE, indicating that the latent variables were both correlated with and distinct from each other. Therefore, the discriminant validity of the latent variables was good.

**Table 3:** Discriminant validity.

Variables	1	2	3	4	5
Environmental perception	0.51				
Positive emotions	0.53**	0.61			
Educational function	0.47**	0.58**	0.54		
Cultural identity	0.42**	0.65**	0.62**	0.55	
Spiritual transformation	0.40**	0.62**	0.66**	0.69**	0.55

** p< 0.01.

### Analysis of structural model: Hypothesis testing

**Fig 2:**
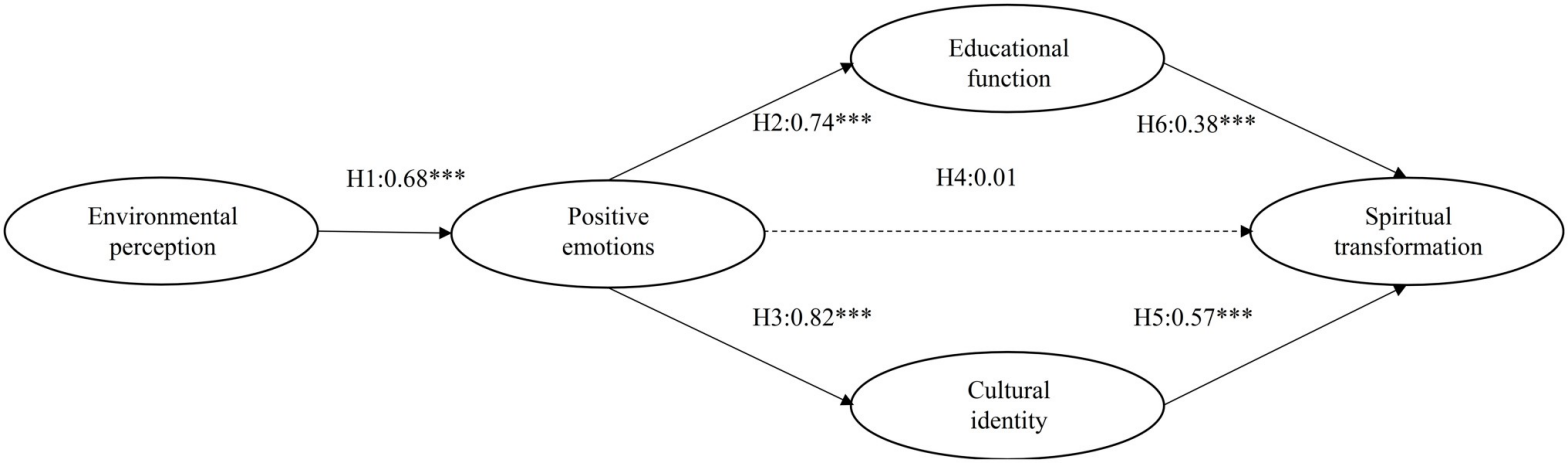
Results of structural equation modelling.

We tested the good fit of our research model using Amos software (v. 20; IBM SPSS, Inc). The model showed a good overall fit, with the following fit indices: χ2 = 402.352, df = 129, CFI = 0.914> 0.9, and RMSEA = 0.077< 0.08. The proposed structural model was tested using structural equation modelling (Table 4). The estimated path coefficients are shown in Fig 2. H1 holds for a significantly positive effect of environmental perception on tourists’ emotional arousal (β = 0.68, p<0.001), H2 holds for a significantly positive effect of positive emotions on the educational function of red tourism (β = 0.74, p<0.001), and H3 holds for a significantly positive effect of positive emotions on red cultural identity (β = 0.82, p<0.001). Positive emotions do not have a significant effect on individuals’ spiritual transformation (β = 0.01, p = 0.933); thus, H4 does not hold. Red cultural identity has a significantly positive effect on individuals’ spiritual transformation (β = 0.57, p<0.001); thus, H5 holds. Red tourism’s educational function has a significantly positive effect on individuals’ spiritual transformation (β = 0.38, p<0.001); thus, H6 holds. In summary, all of the hypotheses are supported except for H4.

**Table 4:** Results of structural equation modelling.

Hypothesis	β	p	Results
*H1*: Environmental perception → Positive emotions	0.68	***	Supported
*H2*: Positive emotions → Educational function	0.74	***	Supported
*H3*: Positive emotions → Cultural identity	0.82	***	Supported
*H4*: Positive emotions → Spiritual transformation	0.01	0.933	Not supported
*H5*: Cultural identity → Spiritual transformation	0.57	***	Supported
*H6*: Educational function → Spiritual transformation	0.38	***	Supported

*** p< 0.001.

### Mediation effect

To more deeply explore the inner mechanism of spiritual transformation, we examined the mediating effects of educational function and cultural identity between positive emotions and spiritual transformation. We used a bootstrap confidence interval (CI) estimation method to estimate the intervals, with a sample size of 5,000 and a 95% CI (Table 5).

**Table 5:** Results of mediation tests.

Path	Estimation	SE	Bias-corrected 95% CI		p
			Lower	Upper	
Positive emotions → Educational function → Spiritual transformation	0.71	0.07	Total effect		
			0.55	0.84	0.001
	0.36	0.13	Direct effect		
			0.15	0.62	0.001
	0.35	0.08	Indirect effect		
			0.21	0.52	0.001
Positive emotions → Cultural identity → Spiritual transformation	0.71	0.07	Total effect		
			0.54	0.83	0.001
	0.17	0.15	Direct effect		
			-0.11	0.46	0.242
	0.54	0.12	Indirect effect		
			0.35	0.84	0.001

In our analysis of the mediating effect of the educational function, the estimated coefficient of the total effect of positive emotions on spiritual transformation was 0.66 (p = 0.001) with a CI (0.55–0.84) that did not contain 0, indicating a significant total effect of positive emotions on spiritual transformation. The estimated coefficient of the indirect effect of positive emotions on spiritual transformation was 0.35 (p = 0.001) with a CI (0.21–0.52) that did not contain 0, indicating a significant indirect effect of positive emotions on spiritual transformation. In addition, the significant direct effect of positive emotions on spiritual transformation (effect coefficient, 0.36; p = 0.001; CI did not contain 0) indicates that the educational function partially mediates the relationship between positive emotions and spiritual transformation.

In our analysis of the mediating effect of cultural identity, the estimated coefficient of the total effect of positive emotions on spiritual transformation was 0.71 (p = 0.001) with a CI (0.54–0.83) that did not contain 0, indicating that the total effect of positive emotions on spiritual transformation was significant. The estimated coefficient of the indirect effect of positive emotions on spiritual transformation was 0.54 (p = 0.001) with a CI (0.35–0.84) that did not contain 0, indicating a significant indirect effect of positive emotions on spiritual transformation. In contrast, the direct effect of positive emotions on spiritual transformation was not significant (effect coefficient, 0.17; p = 0.242; CI contains 0); therefore, cultural identity plays a fully mediating role between positive emotions and spiritual transformation.

In summary, positive emotions can contribute to spiritual transformation through the mediating roles of educational function and cultural identity, and cultural identity plays a fully mediating role.

## Conclusions, discussion, and implications

### Conclusions and discussion

Taking the Red Army Long March Xiangjiang Battle Memorial Park as an example of a red tourism site, this paper followed the logic of SOR theory to explore the influential relationships between environmental perception, positive emotion, educational function, cultural identity, and spiritual transformation from an empirical perspective. After structural equation model testing the mediating effects of educational function and cultural identity, we obtained the following conclusions.

Environmental perceptions influence positive emotions; therefore, the stronger the tourists’ environmental perceptions, the stronger their positive emotions. According to SOR, environmental stimuli may cause changes in the state of the organism, which is confirmed in this study. Previous studies have shown that dark tourism experience can affect tourists’ mood [6], but the results of this study confirm that such a phenomenon also exists in red tourism. The red elements exhibited in the Xiangjiang Battle Memorial Hall, such as guns, lists of the fallen, and photos of the battle, create a special environmental atmosphere that differs from general leisure and entertainment tourism because of their visual stimulation and deep impression on visiting tourists. Through their depiction of war conditions and reproduction of objects, tourist destinations bring tourists closer to China’s revolutionary history and promote their positive emotions.

Tourists’ positive emotions influence educational function and cultural identity. That is, the stronger their positive emotions, the stronger the educational function of red tourism and the stronger tourists’ cultural identity. Based on the SOR model, an organism factor may trigger response. Previous research has shown that the emotions generated by visitors to memorials can have an impact on tourists’ behaviour [31], and the dark tourism experience will enhance national identity [45, 46]. Furthermore, war memorial sites can be effective in nurturing and enhancing visitors’ sense of national identity, and are also a typical form of patriotic education [47]. The results of this paper confirm this point, and also expand on previous researches that suggest the red tourism experience has an impact on visitors’ education while on their cultural identity as well. We found that positive tourists’ emotions at red tourism sites, such as sadness and shock, invariably have a significant influence on tourists’ behaviour. In the exhibition hall, the presented historical memories about the Red Army’s Long March stimulated tourists’ senses to help them understand how the revolutionary heroes led the Chinese people to victory in the war. The tourists deeply experienced the indomitable, sacrificial spirit of these revolutionary heroes, which generated a series of positive emotions that not only enriched their understanding of history through the educational value of the red tourism sites but also deepened their sense of red cultural identity.

Although positive emotions did not have a significant direct effect on the spiritual transformation of individual tourist, further research provided the following results. Two pathways for positive emotions culminate in spiritual transformation through educational functions and cultural identity: positive emotions——educational functions——spiritual transformation, positive emotions——cultural identity——spiritual transformation. Some researches have pointed out that dark tourism can promote destination residents to excavate the spiritual culture of disasters [45]. War tourism can effectively cultivate and enhance the national pride of tourists through commemorative festivals and excursions, which is an important means to carry forward the national spirit and establish patriotic consciousness [47]. Different from previous researches, this study concludes two new paths of red tourism’s influence on tourists’ spirit.

The mediating role played by educational function and cultural identity between positive emotions and spiritual transformation is reflected in two main ways: (i) educational function plays a partially mediating role between positive emotions and spiritual transformation; (ii) cultural identity plays a fully mediating role between positive emotions and spiritual transformation. In other words, tourists’ positive emotions can be inspired by the unique political environment atmosphere at the red tourism sites during their visit. These emotions are internalized in the process of spiritual transformation, which must be realized through red tourism’s celebration of cultural identity. The fully mediating role of cultural identity indicates that cultural identity is a very important factor in tourists’ spiritual transformation, which is influenced by emotions and other factors, in line with the SOR model [23]. Tourists are guided to appreciate the red spirit, carry forward red traditions, strengthen their mission, and take the spirit of the Red Army’s Long March into the new era. Red culture is often “silent” in its indoctrination of individual tourists during their visits to revolutionary monuments. However, these tourists can incorporate the spiritual power of red culture into their own spiritual consciousness and behaviour through the indoctrination of red tourism and the subsequent enhancement of their cultural identity. This result confirms the findings of earlier studies exploring tourists’ internalization of red cultural values during red tourism activities [12, 29]. Thus, the protection of historical sites should be strengthened, the value of red tourism sites should be explored, and the ability of red tourism sites to edify red culture should be enhanced. Tourist experiences should be innovated through the design of more stimulating activities and enriched red cultural content at tourism sites to improve education on and the internalization of red culture.

Overall, our study findings show that red tourism activities are the most direct and effective way for tourists to engage with the revolutionary spirit at red tourism sites. The of tourism experience process engages with tourists’ understanding and perceptions in addition to creating emotional breakthroughs, during which tourists can better understand and accept red cultural knowledge and content and can experience the spirit of their revolutionary ancestors’ hard work and dedication through feeling, transposition, imagination, and empathy. This process serves the educational function of red tourism, deepens tourists’ sense of cultural identity, and facilitates the internalization of China’s revolutionary spirit at the spiritual level.

### Management inspiration

Managers of scenic tourism sites, such as red tourism memorials, should focus on mobilizing tourists’ positive emotions. Digital technique can be used to optimize red tourism sites, such as recreating revolutionary scenes from the past to promote immersive experiences for tourists. For example, some popular red tourism activities include “scripted kill” and “immersion kill,” where tourists can experience the red cultural atmosphere. Activity designs could include richer digital elements to simulate the war conditions during the revolutionary period; therefore, tourists would have a deeper impression of the past events through stimulating their senses. Thus, tourists’ feelings about their revolutionary past could be deepened further. Exhibitions should also include a timeline with more explanatory panels showcasing the old photos and bloody stories of the Red Army’s Long March. In addition, a tour guide could provide guided explanations and interpretations of historical events to drive the tourists’ emotional responses.

Zuo et al. found that if the residents of red tourism sites establish trust in the government, they will support the further development of red tourism sites [48] and promote the revolutionary red culture. However, red tourism sites should stimulate tourists’ positive emotions toward the heroic revolutionaries’ deeds and appropriately stimulate negative emotions in people with counterrevolutionary and immoral examples to prevent emotional numbness. When educators edify red culture to tourists, they should focus on activities that comprehensively improve students’ multidimensional literacy through the rich context of red tourism sites, where experiential learning becomes more effective [49]. In addition, it is necessary to promote the organic combination of red tourism activities and ideological red cultural education to increase the infectious power of red culture on tourists. In this way, tourists can broadly form red cultural identities by consciously internalizing the external stimuli of red cultural concepts during red tourism activities.

### Innovations, limitations, and future research

This study identified the mediating roles of educational function and cultural identity in positive emotions to spiritual transformation. We defined the concept of spiritual transformation and derived two paths to spiritual transformation through empirical research, thus demonstrating the value of red tourism activities. The domestic and international literature on red tourism and dark tourism is comprehensive. Most red tourism studies focus on certain effects of red culture on tourists, such as the construction of red cultural memory through providing tourists with experiences of a red cultural atmosphere [50], the stimulation of their emotions to communicate the spiritual meaning of red tourism sites [6], the influence of red cultural atmosphere on tourists’ political and national identities [14], and values and identity [15]. However, few scholars have studied the influential pathways of red tourism activities on tourists’ spiritual experiences. Based on the findings from these studies, this study constructed two value pathways for spiritual transformation in red tourism sites. These findings provide a breakthrough for the theoretical research literature.

Although studies of mechanisms for spiritual transformation have shown that emotions have two pathways to influence spiritual transformation, differences in value pathways may be observed because the questionnaire used in this study was distributed at representative revolutionary tourism sites in Guangxi. This study did not systematically synthesize findings from red tourism sites such as war sites and the hometowns of revolutionary heroes. Therefore, future studies could explore the differences in value pathways for different types of red tourism sites in addition to the value direction of red tourism activities. Tourists’ motives for participating in red tourism activities may differ from those of leisure-oriented tourists [51, 52]; therefore, future studies could investigate the differences in the value pathways of tourists with different motives. In addition, the theoretical elaboration of spiritual transformation in red tourism-related research has not been specifically defined or extensively researched. Therefore, there may be issues with the undefined scale of influence. Future studies could explore the development of a spiritual value scale to enrich research on the spiritual value of red tourism sites.

## Supporting information

S1 Data (PDF)
