# Supplementary material for: Can red tourism lead to spiritual transformation? Evidence from tourists visiting the Red Army Long March Xiangjiang Battle Memorial Park
Source: PLoS One. 2023 Jul 7;18(7):e0280920. doi: 10.1371/journal.pone.0280920 (PMC10328324; doi:10.1371/journal.pone.0280920)
Supplement: S1 Data — (PDF) [file pone.0280920.s001.pdf]

No.	Environmental perception1	Environmental perception2
1	4	7
2	5	4
3	6	4
4	6	6
5	7	6
6	7	7
7	5	7
8	5	6
9	7	7
10	7	7
11	6	6
12	5	6
13	6	7
14	6	5
15	7	7
16	7	5
17	6	6
18	6	7
19	7	7
20	5	7
21	5	7
22	6	6
23	7	5
24	5	4
25	7	6
26	7	6
27	6	7
28	5	7
29	7	7
30	7	7
31	5	5
32	5	4
33	6	6
34	7	6
35	6	6
36	4	6
37	6	7
38	7	5
39	7	7
40	7	7
41	5	6
42	6	5
43	7	7
44	7	4
45	2	2
46	5	5
47	4	5
48	6	7
49	6	5
50	5	5
51	4	6
52	4	4
53	6	5

54	5	5
55	5	4
56	1	1
57	7	7
58	5	5
59	4	5
60	5	5
61	5	6
62	6	6
63	6	6
64	7	7
65	7	7
66	6	6
67	6	6
68	5	5
69	4	4
70	6	6
71	7	7
72	6	7
73	6	6
74	3	5
75	4	5
76	7	7
77	5	5
78	7	7
79	6	6
80	5	6
81	3	5
82	7	7
83	7	7
84	5	5
85	7	7
86	5	5
87	6	7
88	7	6
89	6	6
90	6	6
91	6	6
92	4	6
93	6	6
94	5	5
95	6	6
96	6	7
97	7	7
98	7	7
99	4	4
100	5	7
101	7	7
102	5	7
103	6	6
104	5	6
105	4	7
106	6	7
107	7	7

108	5	5
109	6	6
110	7	7
111	7	6
112	7	7
113	7	7
114	5	5
115	7	7
116	6	6
117	6	7
118	7	7
119	7	7
120	6	6
121	7	7
122	6	5
123	7	7
124	7	7
125	6	6
126	5	5
127	6	6
128	6	6
129	6	6
130	5	6
131	7	7
132	7	4
133	7	7
134	7	7
135	1	7
136	7	7
137	7	7
138	7	7
139	7	7
140	7	7
141	7	7
142	3	4
143	4	5
144	6	6
145	6	7
146	1	6
147	6	6
148	6	7
149	6	5
150	7	6
151	5	6
152	7	7
153	7	6
154	7	7
155	4	5
156	5	5
157	7	6
158	7	7
159	5	5
160	4	5
161	7	6

162	7	6
163	4	6
164	6	7
165	3	4
166	6	5
167	7	7
168	5	5
169	6	7
170	6	6
171	5	6
172	7	6
173	6	6
174	7	7
175	7	6
176	7	7
177	6	7
178	7	7
179	7	7
180	6	6
181	7	7
182	7	7
183	5	5
184	5	5
185	7	7
186	7	7
187	6	7
188	6	5
189	4	7
190	2	4
191	6	6
192	6	6
193	7	7
194	7	7
195	6	6
196	7	6
197	5	5
198	6	6
199	7	7
200	6	6
201	6	6
202	7	7
203	6	6
204	5	7
205	7	7
206	6	7
207	5	5
208	7	7
209	6	6
210	7	7
211	4	4
212	7	7
213	6	5
214	5	6
215	5	6

216	6	6
217	6	7
218	5	7
219	7	6
220	7	7
221	6	6
222	7	6
223	6	7
224	5	6
225	7	7
226	6	5
227	4	7
228	7	6
229	7	6
230	7	7
231	5	5
232	5	6
233	5	6
234	7	7
235	4	5
236	7	6
237	7	7
238	4	4
239	7	7
240	6	5
241	5	7
242	6	5
243	7	7
244	5	3
245	5	5
246	7	7
247	7	7
248	7	7
249	7	7
250	6	6
251	7	7
252	5	6
253	6	6
254	6	6
255	5	6
256	6	6
257	7	7
258	7	7
259	5	3
260	2	7
261	2	6
262	5	7
263	6	4
264	7	4
265	6	4
266	5	4
267	5	5
268	7	6
269	5	4

270	6	5
271	7	7
272	5	5
273	6	6
274	7	7
275	7	7
276	7	6
277	5	5
278	5	5
279	7	6
280	5	6
281	7	7
282	4	4
283	6	6
284	7	7
285	6	6
286	7	7
287	6	7
288	5	6
289	5	5
290	6	7
291	6	7
292	5	5
293	6	6
294	4	4
295	6	6
296	5	6
297	5	6
298	7	6
299	3	6
300	6	6
301	3	3
302	2	2
303	3	2
304	4	6
305	5	6
306	5	4
307	7	7
308	7	6
309	7	7
310	7	7
311	6	6
312	7	7
313	5	6
314	4	7
315	7	7
316	5	6
317	6	6
318	6	5
319	6	5
320	4	7
321	7	7
322	7	7
323	6	7

324	7	7
325	6	7
326	4	7
327	7	7
328	7	7
329	6	5
330	7	4
331	6	7
332	4	7
333	7	4
334	2	7
335	6	5
336	3	2
337	6	6
338	7	6
339	7	5
340	4	7
341	5	6
342	7	7
343	6	7
344	7	7
345	7	7
346	7	7
347	7	7
348	6	6
349	7	7
350	7	5
351	7	6
352	7	7
353	5	7
354	6	6
355	7	7
356	7	7
357	6	6
358	5	5

Environmental perception3	Environmental perception4	Positive emotions1
7	4	7
5	4	7
3	1	5
6	6	7
6	7	7
6	4	7
6	5	6
6	4	6
6	3	7
3	4	7
7	7	7
7	6	7
5	6	7
7	7	6
5	6	7
6	7	7
6	7	7
7	5	7
7	6	7
5	7	7
7	4	6
7	7	7
7	7	5
6	7	5
7	5	5
7	6	5
5	7	7
7	5	4
6	6	7
7	5	7
5	4	6
4	5	7
7	5	7
6	4	5
5	6	7
4	6	6
6	6	5
4	7	7
7	7	7
5	7	7
5	7	7
4	3	7
6	7	7
7	7	7
6	5	5
6	3	5
6	5	7
5	2	7
6	5	6
6	6	5
7	6	7
4	4	5
6	3	5

5	5	5
5	5	5
1	1	7
7	7	6
5	5	5
4	5	4
5	5	7
7	6	6
6	6	7
6	6	5
7	7	7
7	7	7
5	6	6
6	6	6
5	5	5
4	4	4
6	6	6
7	7	6
6	1	7
6	6	6
5	6	6
6	7	5
7	7	7
6	6	6
7	7	7
5	6	7
6	6	5
5	5	5
7	7	7
6	7	7
5	5	5
6	7	7
5	5	6
7	7	6
6	5	7
6	7	7
6	6	6
6	7	7
5	5	5
6	6	6
6	6	7
6	5	7
7	7	6
7	7	7
7	7	7
6	6	6
7	7	6
7	7	7
7	7	7
6	6	6
6	6	6
7	5	5
5	5	7
7	7	7

5	5	5
6	6	6
7	7	7
7	7	7
7	7	7
7	7	7
5	5	7
7	7	7
6	6	6
6	6	7
7	7	7
6	6	6
6	6	6
7	6	7
7	5	7
6	7	7
6	6	7
6	6	6
5	5	7
6	6	6
6	6	6
6	6	6
6	6	6
7	7	6
6	5	7
7	7	7
7	6	7
7	7	7
7	7	7
7	7	7
7	7	7
5	6	7
7	7	7
7	7	7
4	5	5
3	5	6
6	4	7
7	7	7
7	6	6
7	6	7
6	7	7
6	7	6
6	7	5
7	7	7
6	5	6
6	6	6
7	7	7
4	7	6
7	6	6
7	7	7
5	7	7
5	5	6
4	7	7
5	6	7

7	6	7
6	3	7
7	2	7
7	5	6
4	5	5
6	7	7
4	7	6
7	7	7
7	6	6
5	6	7
7	5	6
5	5	5
6	5	7
7	6	6
7	6	7
7	7	7
7	7	7
7	7	7
6	7	7
7	7	7
7	7	7
5	5	6
5	5	6
7	7	7
7	4	7
6	7	7
5	7	7
7	7	7
5	6	5
6	6	7
5	3	7
7	7	7
7	7	7
6	6	7
6	6	6
5	4	5
6	6	7
6	6	6
6	6	7
6	6	6
5	1	7
6	5	7
7	7	7
6	5	5
6	7	6
4	4	6
6	6	7
5	3	7
7	5	7
4	4	4
7	7	5
6	7	7
6	7	6
6	7	5

5	5	7
7	7	5
7	6	7
6	7	7
5	7	7
4	2	6
7	6	7
5	7	7
7	3	5
7	7	7
7	4	7
7	6	6
6	5	7
6	5	7
7	7	7
5	5	5
7	4	6
6	6	7
7	7	6
5	6	6
5	4	6
7	7	7
4	5	5
7	7	7
7	7	7
7	7	6
6	5	3
7	7	7
4	5	6
5	4	5
7	7	7
7	7	7
7	7	7
7	7	7
7	5	7
7	7	7
5	5	6
6	7	6
6	7	7
6	7	6
6	6	6
6	6	6
7	7	7
3	6	4
7	7	7
6	6	6
5	6	7
7	7	6
6	7	6
4	6	7
5	4	4
7	7	4
7	3	2
6	5	7

7	5	5
7	7	7
5	5	6
6	6	6
5	5	7
7	7	6
7	7	7
5	5	4
4	4	5
7	6	6
7	7	7
5	5	7
4	4	6
7	6	7
7	7	7
6	4	7
7	6	7
7	7	7
7	7	5
5	3	6
7	5	7
6	6	7
6	7	7
6	6	7
4	5	6
6	4	7
5	6	7
5	4	6
6	6	7
7	6	7
7	7	7
6	6	4
2	1	1
2	2	7
6	7	4
6	6	7
5	5	7
7	6	7
6	6	7
7	7	7
7	7	7
6	6	7
7	7	7
5	5	7
6	7	7
7	7	7
6	5	6
7	6	6
5	5	5
7	7	7
6	7	7
7	7	7
6	7	7
6	7	7

6	6	7
7	7	7
7	7	7
7	7	7
7	7	7
6	6	6
5	5	7
7	7	7
3	7	7
4	6	7
7	3	5
4	7	6
4	4	5
5	3	5
3	7	5
4	7	6
4	4	7
3	6	2
7	7	7
5	7	7
7	7	7
7	7	7
7	6	7
7	7	7
5	5	7
7	7	6
7	6	6
6	7	6
5	4	5
7	1	7
5	5	6
6	7	5
6	6	7
6	4	7
5	5	5

Positive emotions2	Positive emotions3	Cultural identity1	Cultural identity2
7	7	6	6
6	7	7	7
5	5	6	6
7	7	7	7
7	6	6	7
7	7	7	7
6	6	7	7
7	5	5	6
6	5	6	7
4	7	7	6
7	7	6	6
7	7	7	7
7	7	7	7
7	7	7	7
7	7	7	6
7	7	7	7
7	7	6	6
7	7	7	7
7	7	7	7
6	7	7	7
7	5	7	7
6	7	7	6
7	7	7	5
6	7	7	7
7	7	6	7
6	7	7	6
7	7	7	7
4	6	5	7
7	7	7	6
7	7	7	7
6	4	6	6
6	5	7	6
6	5	7	5
6	6	7	6
7	7	7	7
6	6	6	6
6	5	7	6
7	7	7	7
7	7	7	7
7	7	7	7
7	7	7	7
7	7	7	7
7	7	7	7
7	7	7	6
5	5	7	5
4	5	5	6
7	7	7	7
7	7	7	7
6	7	7	6
7	7	6	7
7	7	7	7
4	4	5	4
3	6	6	3

6	6	7	7
5	4	5	4
7	7	7	7
7	6	7	6
5	5	5	5
5	5	5	5
7	7	7	7
7	7	7	7
7	7	7	7
6	5	7	7
7	7	7	7
7	7	7	7
6	6	7	6
6	6	6	6
5	5	5	5
4	4	4	4
6	6	7	7
6	6	6	6
7	6	7	7
7	7	6	7
7	7	7	7
6	7	6	6
7	7	7	7
6	6	6	6
7	7	7	7
7	6	7	7
6	6	5	6
5	5	5	5
7	7	7	7
7	7	7	7
5	5	5	5
7	7	7	7
6	6	7	7
7	7	7	7
7	6	6	7
6	6	7	7
7	7	7	7
7	7	7	7
5	6	5	5
6	6	6	6
7	7	7	7
7	7	7	5
7	7	7	7
7	7	7	7
7	7	7	7
6	6	6	6
6	5	7	7
7	7	7	7
7	7	7	7
6	6	6	6
6	7	7	7
3	7	7	7
7	7	7	5
7	7	7	7

5	5	5	5
6	6	6	6
7	7	7	7
7	7	6	6
7	7	7	7
7	7	7	7
7	5	7	7
7	7	7	7
6	6	6	6
7	7	7	7
7	7	7	7
7	7	7	7
6	6	7	7
7	7	7	7
7	7	7	7
7	7	7	7
7	7	7	7
6	6	6	6
7	7	7	7
6	6	6	6
6	6	6	6
6	6	6	6
7	5	6	7
7	7	7	7
7	7	7	7
7	7	7	7
7	7	7	7
7	7	7	7
7	7	7	7
7	7	7	7
7	7	7	7
7	7	7	7
7	7	7	7
7	7	7	7
4	4	5	6
5	6	5	4
7	7	7	7
7	7	7	7
6	7	6	7
7	6	7	7
7	7	7	7
5	6	6	5
6	6	7	7
7	7	7	7
7	7	7	7
7	7	7	7
7	7	7	7
7	7	6	4
6	6	7	6
6	7	7	7
7	7	6	6
6	7	7	7
7	7	7	7
7	6	7	6

7	7	7	7
7	7	6	6
7	7	7	7
6	6	7	7
7	7	7	7
7	7	7	7
6	7	7	6
7	7	7	7
7	6	7	7
7	6	7	7
7	7	7	6
5	5	5	5
7	7	7	6
7	7	7	6
7	7	7	7
7	7	7	7
7	7	7	7
7	7	7	7
7	7	7	7
7	7	7	7
7	7	7	7
6	6	7	7
6	6	7	7
7	7	7	7
7	7	7	7
7	7	7	7
7	7	7	7
7	7	7	7
7	4	5	3
7	7	6	7
7	7	7	7
7	7	7	7
7	7	7	7
7	7	7	7
7	7	7	7
6	6	6	6
7	7	7	7
6	6	5	6
7	7	7	7
6	6	7	7
7	7	7	7
7	7	7	7
7	7	7	7
5	1	5	5
7	6	7	6
7	7	7	6
7	7	7	7
7	7	5	5
7	7	7	7
4	4	7	7
5	7	6	6
6	5	5	5
7	7	7	7
5	6	5	6

7	7	7	7
6	7	7	7
6	5	6	6
6	6	7	7
7	7	6	6
6	7	6	7
7	7	6	6
7	7	7	6
4	6	7	5
7	7	7	7
7	6	7	6
7	6	7	6
6	5	7	6
7	6	7	7
7	7	7	7
5	5	7	7
6	7	5	6
7	7	7	7
7	7	7	6
6	7	7	7
7	6	7	6
7	7	7	7
5	5	5	5
7	7	7	7
6	7	7	7
6	6	7	7
5	5	5	5
7	7	7	7
6	6	6	6
5	5	5	5
7	7	7	7
7	7	7	6
7	7	7	7
7	7	7	7
5	5	6	6
7	7	7	7
6	6	6	6
6	7	7	7
7	7	7	7
6	6	7	7
6	6	6	6
6	7	6	6
7	7	7	7
4	2	7	7
5	5	3	6
5	5	2	4
7	2	7	7
5	7	7	6
7	4	5	7
3	4	4	4
4	6	4	7
7	7	7	6
4	5	5	5
5	2	6	6

2	5	5	2
7	7	7	7
6	6	7	7
7	7	6	7
7	7	7	7
7	7	7	7
7	6	7	7
5	5	7	5
4	4	4	5
5	6	6	6
7	7	6	5
7	7	7	7
6	6	6	6
6	7	6	7
7	7	2	7
7	7	7	7
7	7	7	7
7	7	7	6
7	7	7	7
6	6	5	5
7	5	7	7
7	7	7	7
6	6	7	7
7	7	7	7
6	7	6	6
6	5	6	5
5	7	6	6
6	5	7	7
6	5	7	6
7	7	7	7
6	6	7	7
4	4	5	5
1	1	6	6
7	7	6	7
4	4	6	6
7	7	7	7
7	7	6	5
7	7	7	7
7	7	7	7
7	7	7	7
7	7	7	7
7	7	7	7
7	7	7	7
7	7	6	6
7	7	7	7
7	7	7	7
6	6	7	7
7	7	7	7
5	7	5	7
7	7	7	6
7	7	7	7
7	7	7	7
7	7	6	7
7	7	7	6

7	7	7	7
7	7	7	7
7	7	7	7
7	7	7	7
7	7	7	7
7	7	6	4
6	5	5	7
7	7	7	7
7	7	7	7
2	6	7	5
4	3	5	6
2	7	6	5
4	5	6	6
3	2	3	7
5	7	7	5
2	6	4	3
6	5	6	7
3	7	4	6
7	7	7	7
7	7	5	6
7	7	7	7
6	6	6	6
7	7	6	6
7	7	7	7
7	7	7	7
7	6	5	6
7	7	7	7
5	7	7	5
5	7	7	7
7	5	7	5
7	6	7	7
5	3	7	7
7	6	7	6
7	7	7	6
4	6	7	7

Cultural identity3	Cultural identity4	Educational function1
6	7	7
7	7	7
6	6	6
7	7	6
7	6	6
7	7	7
7	7	7
4	5	6
6	7	6
4	7	6
6	7	7
7	7	7
7	7	6
5	6	7
5	7	7
6	7	7
7	7	6
7	7	6
7	7	7
7	7	7
7	7	6
7	6	7
6	7	4
6	7	7
7	5	5
7	5	6
7	7	7
7	7	6
5	5	6
6	7	7
6	5	6
5	7	7
6	7	5
6	7	6
7	7	7
7	7	7
6	7	7
7	7	7
7	7	7
7	6	7
7	7	7
7	7	5
7	7	4
6	7	7
5	7	4
5	5	6
7	7	7
7	7	7
6	7	6
7	7	7
7	7	6
5	3	5
6	4	6

7	7	5
5	5	5
7	7	7
6	6	6
5	5	5
4	4	6
7	7	7
7	7	6
7	7	7
7	7	6
7	7	7
7	7	7
6	7	6
6	6	6
5	5	5
4	4	4
7	7	6
6	6	6
7	6	7
6	7	7
7	7	6
7	7	5
7	7	7
6	6	5
7	7	7
6	7	6
7	7	6
5	5	5
7	7	7
7	7	7
7	7	7
7	7	7
7	7	7
7	7	7
7	7	6
7	7	6
7	7	7
7	7	7
6	6	6
6	6	6
7	7	7
6	6	6
7	7	5
7	7	7
7	7	7
6	6	6
7	7	7
7	7	7
6	5	7
6	6	6
7	7	7
7	7	7
6	6	6
7	7	7

5	5	5
6	6	6
7	7	7
7	6	7
7	7	7
7	7	7
7	7	7
7	7	7
6	6	6
7	7	7
7	7	7
7	7	7
6	6	6
7	7	7
7	7	7
7	7	7
7	7	7
6	6	6
7	6	6
6	7	7
6	6	6
7	7	7
5	6	6
7	7	7
7	7	7
7	7	7
7	6	7
7	7	7
7	7	7
7	7	7
7	7	7
7	6	5
7	7	7
7	7	7
5	5	7
5	5	5
7	7	7
7	7	7
6	7	6
7	7	7
7	7	5
7	5	6
7	7	7
7	7	7
6	6	7
7	7	6
6	6	7
7	7	6
5	5	5
7	7	7
6	6	6
7	7	7
7	7	7
7	7	7

5	7	7
7	6	7
7	7	7
6	6	5
7	7	7
7	7	7
7	7	6
7	7	6
7	7	7
7	7	7
7	7	7
5	5	5
6	7	7
7	7	6
7	7	7
7	7	7
7	7	7
7	7	7
7	7	7
7	7	7
7	7	7
6	7	7
7	7	5
7	7	7
7	7	7
7	7	7
7	7	7
7	7	7
2	3	5
7	7	7
7	7	7
7	7	7
7	7	7
7	7	7
7	6	7
6	6	5
7	7	7
5	6	6
7	7	6
7	7	6
7	7	6
7	7	7
7	7	7
6	4	5
7	5	5
7	6	5
7	7	7
5	5	6
7	7	7
7	7	7
6	7	6
5	5	5
7	7	6
6	6	5

7	7	7
5	7	7
5	7	7
7	7	7
6	6	7
7	7	5
7	7	6
7	6	7
7	6	7
7	7	7
5	6	5
7	6	7
7	7	7
7	5	7
7	7	7
7	7	7
5	7	6
7	7	7
6	7	7
7	7	6
6	7	7
7	7	7
5	5	5
7	7	7
7	7	6
7	7	7
3	3	5
7	7	7
6	6	6
5	5	4
7	7	7
7	7	7
7	7	7
7	7	7
6	7	6
7	7	7
6	6	6
7	7	7
7	7	7
7	7	6
6	6	7
6	7	6
7	7	7
3	6	5
6	6	5
3	6	7
6	5	6
5	6	7
7	6	6
6	7	7
5	5	7
7	6	2
5	6	4
6	3	6

1	4	6
7	7	7
7	7	7
7	6	6
7	7	7
7	7	7
7	7	7
6	5	7
4	5	4
5	6	7
6	4	6
7	7	7
6	6	7
6	6	6
5	6	7
7	7	7
7	7	7
7	7	7
4	6	7
5	6	7
7	7	7
7	7	6
6	6	7
7	7	7
6	5	6
5	7	5
7	6	7
7	7	6
7	7	7
7	7	7
7	7	7
5	5	3
7	6	6
2	7	6
6	6	7
7	7	7
6	5	5
7	7	7
7	7	6
7	7	7
7	7	7
7	7	7
7	7	7
5	6	7
7	7	7
7	7	7
7	7	7
7	7	7
7	7	6
6	7	7
7	7	7
7	7	7
7	7	7
7	7	7

7	7	7
7	7	7
7	7	7
7	7	7
7	7	7
4	6	5
7	5	6
7	7	7
7	7	7
6	1	6
6	6	3
6	2	2
3	6	7
3	7	7
2	2	7
7	7	5
5	7	7
5	7	3
7	7	7
7	6	7
7	7	7
7	7	7
7	7	7
7	7	7
7	7	7
6	7	7
5	7	4
4	4	7
5	5	4
7	7	7
6	6	7
7	7	7
6	7	7
6	6	7
7	5	5

Educational function2	Educational function3	Educational function4
6	6	7
5	5	7
6	6	6
7	7	6
7	7	7
6	5	7
7	7	7
5	5	6
7	5	6
7	7	6
7	6	6
6	5	6
7	5	5
6	5	5
7	7	7
7	6	7
6	7	7
6	6	7
7	7	7
7	6	7
7	7	5
7	6	7
7	4	5
6	7	7
7	7	5
6	6	5
7	7	7
5	5	4
6	7	6
7	7	7
6	5	6
6	5	7
7	5	7
5	5	4
7	7	7
7	7	7
7	5	5
7	5	6
4	7	7
7	6	7
7	7	7
5	4	5
6	3	4
6	7	7
6	4	2
6	6	6
7	6	7
7	6	7
6	5	5
7	7	7
6	6	6
4	3	6
5	3	6

5	6	6
5	4	5
7	7	7
7	7	7
5	5	5
5	4	5
7	7	7
6	6	6
7	7	7
7	6	7
7	7	7
7	7	7
5	7	7
6	6	6
5	5	5
4	4	4
7	7	7
7	7	7
7	7	7
7	7	7
6	6	6
6	5	6
7	6	7
5	5	5
7	7	7
6	5	7
6	6	6
5	5	5
7	7	7
7	7	7
7	7	7
7	6	7
7	7	7
6	6	7
6	6	7
6	7	7
7	7	7
7	6	7
7	6	7
6	6	6
7	7	7
7	6	7
7	5	5
7	7	6
7	7	7
6	6	5
7	7	7
7	7	7
6	6	7
6	6	6
7	7	7
7	6	7
6	6	7
7	7	7

5	5	7
6	6	6
7	7	7
7	7	7
7	7	7
7	7	7
7	7	7
7	7	7
6	6	6
7	7	7
7	7	7
7	7	7
6	6	6
7	7	7
7	7	7
7	7	7
7	7	7
6	6	6
6	6	7
7	7	7
6	6	6
7	7	7
6	7	6
7	7	7
7	7	7
7	7	7
2	7	7
7	7	7
7	7	7
7	7	6
7	7	7
5	6	6
7	7	7
7	7	7
4	6	6
5	5	5
7	6	6
7	7	7
7	6	7
7	7	7
7	7	6
5	7	6
7	7	6
7	7	7
7	7	7
7	5	6
7	7	7
7	7	7
7	4	6
7	7	7
6	6	6
7	7	7
7	7	7
6	6	7

7	7	7
6	7	5
7	6	7
5	5	6
7	6	7
7	7	7
6	6	7
6	7	6
7	7	7
7	7	7
5	5	6
6	6	7
7	7	7
7	6	7
7	7	7
7	7	7
7	7	7
7	7	7
7	7	7
7	7	7
7	7	7
7	6	6
5	5	5
7	7	7
6	7	7
7	7	7
7	6	7
7	7	7
6	7	7
7	7	7
7	7	7
7	7	7
7	7	7
4	5	7
6	7	6
5	5	5
7	7	7
6	6	7
6	6	7
7	7	7
6	7	7
7	7	7
7	7	7
6	5	4
6	5	7
4	4	4
6	6	6
6	6	7
7	7	7
7	7	7
7	7	6
6	6	6
6	6	7
6	6	5

7	7	7
7	7	5
7	6	5
6	6	7
7	7	7
5	5	5
6	6	6
6	7	6
6	5	5
7	7	7
5	4	5
6	7	7
7	6	7
6	6	7
7	7	7
7	7	7
6	6	5
7	7	7
7	6	6
6	6	6
6	7	6
7	7	7
5	5	5
7	7	7
5	4	5
5	5	7
3	3	3
7	7	7
6	6	7
4	4	4
7	7	7
7	7	7
7	7	7
7	7	7
7	7	6
7	7	7
6	6	6
7	7	7
7	6	6
6	6	6
6	6	7
7	7	7
7	7	7
7	2	1
7	2	7
4	5	6
3	4	6
7	3	7
2	3	6
2	1	3
4	7	6
7	4	2
7	7	5
6	7	7

3	2	7
7	7	7
7	7	7
7	6	7
7	7	7
7	7	7
7	7	7
7	5	5
5	4	5
7	6	7
5	6	5
7	7	7
7	7	7
6	7	7
7	5	6
7	7	7
7	7	7
7	7	7
7	7	7
7	7	6
6	5	5
6	7	7
7	7	7
7	7	7
6	6	6
5	6	6
5	7	7
6	7	7
6	7	7
7	6	7
7	6	6
3	3	3
6	7	6
3	3	2
6	6	7
7	4	5
6	6	5
7	7	7
7	7	7
7	7	7
7	7	7
7	6	6
7	7	7
6	6	5
7	6	6
7	7	7
7	7	7
7	6	7
6	6	5
6	5	6
7	6	6
7	7	7
7	6	7
7	7	7

7	7	7
7	6	6
7	7	7
7	7	7
7	7	7
4	6	6
7	5	5
7	6	7
7	7	7
6	4	7
4	6	3
3	4	7
6	7	6
5	7	5
4	7	5
7	5	5
6	5	3
7	3	4
7	7	7
7	6	6
7	7	7
7	7	6
7	7	7
7	7	7
7	7	7
6	7	7
6	6	7
5	6	6
4	6	4
6	6	4
7	5	6
7	7	7
7	4	6
7	7	7
5	5	5

Spiritual transformation1	Spiritual transformation2	Spiritual transformation3
7	7	6
7	7	7
7	7	7
6	6	7
7	7	6
7	6	7
7	7	7
6	5	6
5	7	7
7	7	7
7	7	6
7	5	6
7	7	7
7	7	7
6	7	7
7	7	7
7	7	6
7	7	7
7	7	7
7	7	7
7	7	7
7	7	6
5	5	7
7	7	6
6	5	7
7	6	6
7	7	7
6	4	6
6	6	7
7	7	7
6	5	7
7	6	4
7	5	7
7	6	7
7	7	7
7	7	7
7	7	6
7	7	7
7	7	7
7	7	7
7	7	7
5	5	7
7	5	6
7	7	7
7	7	7
6	5	6
7	7	7
7	7	7
7	6	7
7	7	7
7	6	7
5	3	3
6	4	6

6	6	7
7	7	7
7	7	7
7	7	6
5	5	5
4	5	4
7	7	7
7	7	7
7	7	7
7	7	7
7	7	7
7	7	7
5	6	7
6	6	6
5	5	5
4	4	4
6	6	6
7	7	7
7	7	7
7	7	7
7	7	7
6	7	7
7	7	7
6	6	6
7	7	7
7	7	7
7	7	7
5	5	5
7	7	7
7	7	7
6	7	7
7	7	7
7	7	7
7	7	7
7	7	6
6	7	7
7	7	7
7	7	7
7	7	7
6	6	6
7	7	7
6	7	7
6	7	7
7	7	7
7	7	7
7	7	7
7	7	7
7	7	7
7	7	6
6	6	6
7	6	7
7	7	7
7	6	7
7	7	7

7	7	7
6	6	6
7	7	7
7	6	7
7	7	7
7	7	7
7	7	7
7	7	7
6	6	6
7	7	7
7	7	7
7	7	7
6	6	6
7	7	7
7	7	7
7	7	7
7	7	7
6	6	6
7	7	7
7	7	7
6	6	6
7	7	7
6	7	6
7	7	7
7	7	7
7	7	7
7	5	7
7	7	7
7	7	7
7	7	7
7	7	7
6	7	7
7	7	7
7	7	7
7	7	6
6	5	6
7	7	7
7	7	7
6	6	6
7	7	7
7	6	7
6	7	5
6	6	6
7	7	7
7	7	7
7	7	7
7	7	7
7	7	7
6	6	6
7	7	7
7	7	7
7	7	7
7	7	7
7	7	7

7	6	7
6	7	7
7	7	7
7	7	7
7	7	7
7	7	7
5	5	5
7	7	7
7	6	7
7	7	7
6	7	6
7	7	7
7	6	7
7	7	7
7	7	7
7	7	7
7	7	7
7	7	7
7	7	7
7	7	7
7	7	7
7	7	5
5	6	6
7	7	7
7	7	7
7	7	7
7	7	7
7	7	7
4	4	7
7	7	7
7	7	7
7	6	7
7	7	7
7	7	7
7	6	6
6	6	6
7	7	7
7	6	6
7	6	7
7	6	7
7	7	7
7	7	7
7	7	7
5	4	5
5	6	5
5	6	7
7	6	7
7	7	7
7	7	7
7	7	7
7	6	6
6	7	7
7	7	7
5	6	6

7	7	7
7	6	7
6	6	7
7	6	6
7	6	7
6	7	7
6	6	6
7	7	7
6	6	5
7	7	7
7	6	7
7	7	7
7	7	7
7	6	7
5	7	6
7	7	7
5	7	7
7	5	7
7	7	7
7	7	7
6	7	7
7	7	7
5	5	5
7	7	7
7	7	7
7	6	7
5	5	6
7	7	7
7	6	7
7	5	7
7	7	7
7	7	7
7	7	7
7	7	7
7	7	7
7	7	7
7	7	7
7	6	7
7	6	7
6	6	6
6	7	7
7	6	6
7	7	7
6	5	5
6	6	4
1	6	7
7	5	1
6	1	6
4	6	5
7	6	4
4	5	7
2	6	5
6	7	5
5	6	3

4	3	5
7	7	7
7	7	7
6	6	7
7	7	7
7	7	7
7	7	7
6	5	7
4	4	4
6	7	5
5	6	4
7	7	7
4	4	4
6	7	6
7	6	7
7	7	7
7	7	7
6	6	6
7	7	7
7	7	7
6	7	7
7	7	7
7	6	6
7	7	7
7	7	7
7	5	6
7	7	7
7	7	7
7	6	7
7	7	7
7	6	7
5	6	5
7	7	7
7	2	7
7	6	6
6	7	7
6	6	6
7	7	7
7	7	7
7	7	7
7	7	7
7	7	6
7	7	7
5	7	6
7	7	7
7	7	7
7	7	7
7	7	7
6	6	6
7	5	7
7	7	7
7	7	7
7	7	7
7	7	7

7	7	7
7	7	7
7	7	7
7	7	7
7	7	7
6	6	7
7	7	7
7	7	7
7	7	7
5	3	6
5	4	2
6	5	7
6	2	4
5	4	4
7	7	4
6	6	6
5	6	7
3	4	6
7	7	7
7	7	7
7	7	7
6	6	7
7	7	7
7	7	7
7	7	7
6	7	7
5	6	7
7	7	7
7	7	7
6	7	7
6	7	7
7	7	7
7	7	7
7	6	7
4	5	5

Spiritual transformation4	Gender	Age	Occupation	Income per month(in RMB Yuan)
6	2	1	1	1
7	1	1	1	1
7	2	2	1	1
7	1	1	1	1
6	2	2	1	1
7	2	2	1	1
7	1	2	1	1
5	1	2	1	1
7	1	3	1	3
7	1	2	1	1
7	2	1	1	1
7	2	2	1	1
7	2	1	1	1
7	1	2	1	1
7	2	1	1	1
7	2	1	1	1
6	2	1	1	1
7	2	1	1	1
7	1	1	1	1
7	2	2	1	1
7	2	2	1	1
6	2	2	1	1
7	2	1	1	1
5	2	2	1	2
6	2	2	1	1
7	2	2	1	1
7	2	1	1	1
5	2	2	1	1
7	1	1	1	1
6	2	1	1	1
7	2	1	1	1
7	1	2	1	1
6	2	2	1	1
7	2	2	1	1
7	2	2	1	1
7	2	2	1	1
7	2	1	1	1
7	1	1	1	1
7	1	1	1	1
7	2	1	1	1
7	2	1	1	1
7	1	1	1	1
4	2	2	1	1
7	2	2	1	1
7	1	1	1	1
5	1	2	1	2
7	1	2	1	2
7	2	2	1	1
7	2	2	1	2
7	1	2	1	1
7	2	2	1	1
5	2	2	1	3
5	2	2	1	1

7	2	2	1	2
7	2	2	1	3
7	2	2	1	1
6	2	2	1	3
5	2	2	1	1
5	2	2	1	1
7	2	2	1	1
7	2	2	1	1
7	1	2	1	1
7	1	2	1	1
7	1	3	1	1
7	2	2	1	3
5	1	2	1	1
6	1	2	1	1
5	1	2	1	1
4	1	2	1	1
7	1	2	1	2
7	1	3	1	1
6	1	2	1	1
7	2	2	1	1
7	2	3	1	1
7	2	2	1	1
7	2	2	1	1
6	1	2	1	1
7	2	2	1	1
7	2	2	1	1
7	2	2	1	1
5	2	2	1	1
7	2	2	1	1
7	2	2	1	1
6	2	2	1	1
7	2	2	1	1
7	1	2	1	1
7	2	2	1	1
6	1	2	1	1
7	1	2	1	1
7	2	2	1	2
7	2	2	1	1
7	1	3	1	1
6	2	3	1	1
7	2	2	1	1
7	1	2	1	1
7	1	2	1	1
7	1	2	1	1
7	2	2	1	1
7	1	2	1	1
7	1	2	1	1
7	2	2	1	1
7	2	2	1	1
7	1	1	1	1
7	2	2	1	1
7	2	1	1	1
6	2	1	1	1
7	1	2	1	1

7	2	1	1	5
6	2	2	1	3
7	1	2	1	1
6	1	2	1	1
7	2	1	1	1
7	1	2	1	1
7	2	1	1	1
7	2	1	1	2
6	2	1	1	1
7	1	2	1	1
7	2	2	1	1
7	2	1	1	1
6	1	2	1	1
7	2	1	1	1
7	1	2	1	2
7	2	2	1	1
7	2	1	1	1
6	2	1	1	1
7	2	2	1	1
7	2	1	1	1
6	1	2	1	1
7	1	2	1	1
5	1	2	1	1
7	2	2	1	1
7	1	2	1	1
7	2	1	1	1
7	2	1	1	1
7	1	2	1	1
7	1	1	1	1
7	2	2	1	1
7	2	3	1	1
7	2	1	1	1
7	2	2	1	1
7	2	1	1	1
7	2	4	2	3
5	1	3	2	4
7	1	4	2	4
7	1	4	2	3
7	1	3	2	4
7	1	4	2	4
6	1	4	2	2
6	1	4	2	4
6	1	3	2	5
7	2	4	2	1
6	2	3	2	3
7	2	3	2	3
7	2	2	2	3
7	1	3	2	4
7	1	4	2	5
7	1	4	2	3
7	1	3	2	3
7	1	3	2	3
7	2	3	2	3
7	1	3	2	3

7	1	4	2	4
7	2	3	2	3
7	1	3	2	5
7	2	3	2	3
7	1	3	2	5
7	2	2	2	3
5	1	2	2	3
7	1	3	2	4
7	1	3	2	4
7	2	3	2	3
5	1	3	2	5
7	1	4	2	3
7	1	2	2	3
7	1	3	2	5
7	1	4	2	5
7	2	2	2	2
7	1	4	2	3
7	1	4	2	4
7	1	3	2	5
7	1	3	2	5
7	2	3	2	4
4	1	2	2	5
6	1	3	2	5
7	1	3	2	4
7	2	3	2	2
7	2	3	2	3
7	2	3	2	2
7	1	3	2	3
5	2	4	3	3
7	1	4	3	3
7	1	4	3	3
7	2	4	3	4
7	1	4	3	5
7	2	3	3	2
6	2	3	3	3
6	1	4	3	5
7	1	3	3	4
6	2	3	3	3
7	1	3	3	4
7	1	4	3	3
7	2	4	3	4
7	2	3	3	3
7	1	4	3	2
3	2	4	3	3
6	2	3	3	3
7	1	3	3	4
7	1	4	3	4
7	2	3	3	3
7	1	3	3	4
7	1	4	3	3
7	2	3	3	3
7	1	3	3	5
7	2	4	3	3
5	2	3	3	3

7	1	4	3	4
7	1	3	3	4
7	1	3	3	3
7	2	4	3	4
6	2	3	3	4
7	1	3	3	5
6	1	4	3	3
7	1	3	3	4
5	2	3	3	3
7	1	4	3	4
6	2	4	3	2
7	1	4	3	3
7	1	4	3	4
7	2	3	3	3
7	2	3	3	2
7	2	3	3	3
6	1	3	3	5
7	2	2	3	3
7	2	2	3	4
7	1	3	3	4
6	1	2	3	2
7	2	2	3	3
5	2	3	3	4
7	2	3	3	3
7	2	3	3	5
7	1	3	3	5
5	2	2	3	5
7	2	2	3	4
7	1	2	3	5
5	1	2	3	3
7	2	2	3	3
7	1	3	3	5
7	1	3	3	4
7	1	3	3	4
6	2	2	3	4
7	2	2	3	2
7	2	2	3	5
7	2	2	3	3
7	1	3	3	5
6	1	3	3	4
7	2	2	3	3
6	2	3	3	4
7	1	3	3	5
6	2	4	3	5
5	1	4	3	3
5	2	4	3	2
6	1	3	3	3
6	2	4	3	4
7	1	4	3	3
7	1	3	3	5
7	1	4	3	5
6	1	3	3	4
3	2	3	3	4
5	2	4	3	4

3	1	2	3	3
7	2	3	4	5
7	2	4	4	1
6	2	2	4	3
7	1	4	4	3
7	2	3	4	2
7	2	3	4	2
7	2	3	4	3
4	2	2	4	3
7	2	4	4	4
7	2	3	4	4
7	1	3	4	5
4	2	3	4	2
6	2	3	4	5
7	1	3	4	4
7	2	3	4	3
7	2	4	4	3
6	2	3	4	3
7	2	3	4	3
7	2	4	4	3
7	2	3	4	2
7	1	4	4	4
6	2	4	4	1
7	2	2	4	1
7	2	3	4	1
7	2	3	4	3
7	1	3	4	4
7	2	3	4	3
7	2	3	4	3
7	1	2	4	2
7	2	3	4	1
5	1	2	4	1
7	2	3	4	1
6	2	3	4	3
6	2	3	4	1
7	2	2	4	2
5	2	3	4	3
7	1	3	4	5
7	2	3	4	4
7	2	3	4	5
7	1	4	4	3
7	1	3	4	5
7	1	3	4	1
6	2	4	4	2
7	2	2	4	3
7	2	3	4	4
7	1	2	4	5
7	2	3	4	3
6	2	2	4	4
7	1	4	4	3
6	2	2	4	5
7	1	2	4	4
7	2	2	4	3
7	2	2	4	4

7	1	3	4	5
7	2	3	4	3
7	2	3	4	3
7	2	3	4	3
7	2	3	4	2
5	2	3	4	3
7	2	4	4	3
7	1	4	4	3
7	2	3	4	3
5	1	3	4	5
7	2	3	4	4
6	2	4	4	4
5	1	3	4	3
7	2	3	4	4
4	1	4	4	3
7	1	3	4	4
6	2	4	4	3
5	2	2	4	3
7	2	4	4	2
7	2	2	4	3
7	2	2	4	2
7	2	3	4	3
7	1	3	4	4
7	1	5	5	3
7	1	5	5	3
7	1	5	5	4
7	1	5	5	1
7	1	4	5	4
7	1	2	5	4
5	1	5	5	3
7	1	4	5	2
7	1	5	5	2
6	1	4	5	1
6	2	2	4	4
5	2	1	1	1

Education	Political status
1	3
1	5
3	3
2	5
3	3
1	3
2	3
3	3
3	2
3	3
3	3
4	1
2	3
4	2
1	5
2	3
1	5
1	5
2	3
3	3
3	3
3	3
3	2
2	1
3	2
3	3
2	3
3	2
1	3
2	5
1	5
3	3
3	3
3	1
3	2
3	3
1	5
1	5
1	5
1	5
1	5
1	5
3	1
3	3
3	5
4	3
4	3
3	3
3	3
3	3
3	3
3	1
3	1

3	3
3	3
3	3
3	2
3	3
3	3
3	3
3	3
3	3
3	3
3	3
3	3
3	3
3	3
3	3
3	3
3	3
4	5
4	1
4	3
4	3
4	3
4	3
4	3
4	3
4	3
4	3
4	1
4	3
4	2
4	3
4	3
4	3
4	2
4	3
4	3
4	1
4	3
1	5
4	1
4	3
4	3
3	3
3	1
4	1
4	3
4	1
4	1
4	1
3	5
3	5
2	3
3	5
3	3

3	5
3	2
2	3
3	5
3	3
1	1
3	3
3	3
2	5
3	3
3	5
3	5
3	5
3	3
3	3
3	3
3	3
3	5
3	5
3	5
3	2
3	5
3	3
3	1
3	5
3	5
2	5
3	3
2	3
2	5
4	3
2	5
3	5
2	3
3	2
3	2
3	1
2	1
3	1
3	5
3	1
3	1
2	1
3	1
3	5
3	1
2	3
3	1
2	1
3	1
3	5
3	1
3	1
3	1

3	1
3	5
4	1
4	1
4	1
4	1
3	1
4	1
4	1
3	1
4	1
3	1
3	3
3	5
4	1
3	3
3	1
3	1
3	1
4	1
4	5
3	3
3	2
3	1
3	5
3	3
3	5
4	3
2	5
1	2
2	1
2	1
4	4
2	5
2	5
2	1
4	2
3	1
2	1
2	1
2	1
1	3
2	1
3	1
3	1
3	5
3	1
2	5
4	1
2	5
2	3
3	5
3	5
3	5

3	5
2	5
3	3
2	5
3	5
3	5
3	1
3	5
2	3
3	5
2	3
3	5
3	5
2	3
2	5
2	5
3	5
3	3
3	1
3	5
3	3
3	3
3	5
3	5
4	3
4	3
3	2
3	3
3	3
3	3
3	3
2	5
3	3
2	3
3	5
2	5
2	5
3	3
4	1
3	5
3	5
3	3
2	5
3	3
4	5
4	5
3	3
3	2
2	3
4	1
4	5
3	1
2	1
4	5

3	3
2	5
1	5
2	5
1	5
2	5
2	5
1	5
4	1
3	5
3	5
1	1
2	1
3	5
3	5
2	3
2	5
1	5
3	1
1	5
2	5
2	5
2	3
2	5
1	5
2	5
2	5
1	5
2	5
2	5
1	5
3	3
1	5
3	5
1	5
3	3
1	5
3	5
2	5
3	5
1	1
1	5
4	1
1	5
1	5
4	5
3	1
3	5
3	5
2	5
3	1
2	5
2	5
1	5

2	1
2	5
1	5
1	5
1	5
3	5
1	5
1	1
2	1
3	2
3	1
3	5
2	3
2	5
2	3
3	5
3	3
2	3
1	5
2	5
3	3
2	5
2	5
2	1
3	1
3	1
1	1
2	5
2	1
1	1
2	5
1	5
1	1
3	5
2	3
